# First gene-ontology enrichment analysis based on bacterial coregenome variants: insights into adaptations of *Salmonella* serovars to mammalian- and avian-hosts

**DOI:** 10.1186/s12866-017-1132-1

**Published:** 2017-11-28

**Authors:** Arnaud Felten, Meryl Vila Nova, Kevin Durimel, Laurent Guillier, Michel-Yves Mistou, Nicolas Radomski

**Affiliations:** Université PARIS-EST, Anses, Laboratory for food safety, Maisons-Alfort, France

**Keywords:** Bacterial genomics, Bacterial fixed variants, Gene-ontology enrichment analysis

## Abstract

**Background:**

Many of the bacterial genomic studies exploring evolution processes of the host adaptation focus on the accessory genome describing how the gains and losses of genes can explain the colonization of new habitats. Consequently, we developed a new approach focusing on the coregenome in order to describe the host adaptation of *Salmonella* serovars.

**Methods:**

In the present work, we propose bioinformatic tools allowing (i) robust phylogenetic inference based on SNPs and recombination events, (ii) identification of fixed SNPs and InDels distinguishing homoplastic and non-homoplastic coregenome variants, and (iii) gene-ontology enrichment analyses to describe metabolic processes involved in adaptation of *Salmonella enterica* subsp. *enterica* to mammalian- (*S.* Dublin), multi- (*S.* Enteritidis), and avian- (*S.* Pullorum and *S.* Gallinarum) hosts.

**Results:**

The ‘VARCall’ workflow produced a robust phylogenetic inference confirming that the monophyletic clade *S.* Dublin diverged from the polyphyletic clade *S.* Enteritidis which includes the divergent clades *S.* Pullorum and *S.* Gallinarum (i). The scripts ‘phyloFixedVar’ and ‘FixedVar’ detected non-synonymous and non-homoplastic fixed variants supporting the phylogenetic reconstruction (ii). The scripts ‘GetGOxML’ and ‘EveryGO’ identified representative metabolic pathways related to host adaptation using the first gene-ontology enrichment analysis based on bacterial coregenome variants (iii).

**Conclusions:**

We propose in the present manuscript a new coregenome approach coupling identification of fixed SNPs and InDels with regards to inferred phylogenetic clades, and gene-ontology enrichment analysis in order to describe the adaptation of *Salmonella* serovars Dublin (i.e. mammalian-hosts), Enteritidis (i.e. multi-hosts), Pullorum (i.e. avian-hosts) and Gallinarum (i.e. avian-hosts) at the coregenome scale. All these polyvalent Bioinformatic tools can be applied on other bacterial genus without additional developments.

**Electronic supplementary material:**

The online version of this article (10.1186/s12866-017-1132-1) contains supplementary material, which is available to authorized users.

## Background

Recent advances in bacterial genomics aim to identify small insertions/deletions (InDels) of the coregenome [1] in addition to single nucleotide polymorphisms (SNPs) [2] and offer a unique opportunity to perform the first gene-ontology enrichment analysis based on sensitive and specific variants previously identified according to inferred bacterial clades [3]. These variants correspond to spontaneous mutations or recombination evens [4] which are usually identified thanks to the detection of SNP hotspots [5]. Rare genetic variants near the final leaves of the phylogeny [6] are described as unstable [7] or transient variants [8], in opposite to fixed variants of the branches which are passed on to descendants in the absence of recombination events [9].

Representing several tens of Bioinformatics tools developed since the beginning of the twenty-first century, the gene-ontology enrichment analysis allows identification of relevant biological processes among large lists of genes [10]. Covering Eukaryotic and Prokaryotic organisms [11], the gene-ontology enrichment analysis can be applied on lists of annotated genes or variants [12]. Based on several annotation databases [13], such as Gene Ontology (GO) [14] where the GO-terms form a directed acyclic graph (DAG) organized as three independent ontologies of GO-terms, namely biological process (BP), molecular function (MF), cellular component (CC), different kinds of gene-ontology enrichment analyses must be distinguished. The enrichment likelihood may indeed be calculated using Chi-square, Fisher’s exact test, Binomial probability based on pre-selected interesting gene lists (i.e. a singular enrichment analysis), entire genes with associated experimental values (i.e. a gene set enrichment analysis) [10], or using hypergeometric distribution based on the approaches term-for-term (i.e. a modular enrichment analysis excluding dependencies between the GO-terms) and parent-child (i.e. a modular enrichment analysis including dependencies between the GO-terms) [15].

With around 80 million cases annually, *Salmonella enterica*, especially *Salmonella enterica* subsp. *enterica* serovars, is the main cause of foodborne gastroenteritis [16]. Host adaptation of *Salmonella* is related to acquired pathogenicity islands (PAIs) called *Salmonella* pathogenicity islands (SPIs), and genes involved in intestinal phase of infection, colonization of deeper tissues, and expansion in host range, successively [17]. Several studies tend to theorize that the gains of large genetic elements by horizontal gene transfer (e.g. plasmids, transposons and phages) would expand the host range of *S. enterica* subsp. *enterica* serovars, especially those causing severe human infections (e.g. *S*. Typhimurium and *S*. Enteritidis), while the reduction of host range (e.g. *S*. Typhi in humans, *S*. Dublin in cattle and humans, *S*. Gallinarum in avian hosts) would mainly be explained by the losses of genes due to large deletions and accumulation of pseudogenes (i.e. start/stop gained codons and/or frameshift mutations) [18]. The few genomic approaches aiming for description of host adaptation between [19–22] or into *Salmonella* serovars [4, 23–25], focused on the accessory genome which refers to gains and losses of large genetic elements [26].

The evolution of serovars, as Agona, Dublin, Hadar, Heidelberg, Javiana, Kentucky, Newport, Saintpaul, Schwarzengrund, Virchow, Weltevreden, Choleraesuis, Enteritidis, Gallinarum, Paratyphi A, Paratyphi B, Paratyphi C, Typhi and Typhimurium, seems to be highly mediated by losses of coding sequences which lead to functional reduction, and horizontal acquisitions of plasmid and phage sequences, hypothetically under regulation of a defense mechanism against exogenous invading sequences, particularly through clustered regularly interspaced short palindromic repeats (CRISPR) [20]. With the exception of structural RNA genes and transposable elements, large stable duplications are rare for *Salmonella* and other *Enterobacteriaceae* (e.g. locus *ccm* of 7.5 kb in S. Typhimurium) [27], and many *S. enterica* serovars, as Abortusequi, Abortusovis, Choleraesuis, Dublin, Enteritidis, Gallinarum, Pullorum, Sendai, Paratyphi C, and Typhimurium, are known to harbor different virulence plasmids [21] heterogeneous by size (50–90 kb) but all sharing five genes (i.e. *spvR*, *spvA*, *spvB*, *spvC*, and *spvD*) in the 7.8-kb *spv* region (*Salmonella* plasmid virulence) which are required for bacterial multiplication in the reticulo-endothelial system [28] and cytopathic effect in the human macrophages [29].

Known as a common serovar involved in domestically acquired foodborne illness in humans, *S*. Enteritis is introduced in the food chain by products from diverse hosts [22]. In contrast, foodborne illness in humans caused by *S*. Dublin is mainly due to contaminated cow’s milk and cheese [30], while *S*. Gallinarum [31] and *S*. Pullorum [22] infect avian-hosts. According to a microarray study dividing *S*. Enteritidis into two major clades [19], a recent phylogenetic inference based on SNPs of the coregenome (reference genome: *S*. Enteritidis strain P125109, NC_011294.1) [22] demonstrated that *S*. Dublin and *S*. Enteritidis diverged from a most recent common ancestor (MRCA), and that *S*. Enteritidis was constituted of two clades (e.g. classical and unclassical), one of them being the origin of the avian-adapted serovars *S*. Gallinarum and *S*. Pullorum [22]. Focusing on gene acquisition and functional gene losses explaining host specialization of these bacterial pathogens, Langridge et al. emphasized that evolution of *Salmonella* pathogenicity is strongly associated with the acquisition of SPIs [22], especially SPI-6 and SPI-19 which are mobile genetic elements encoding type VI secretion systems (T6SSs) [31]. While T6SSs in SPI-19 of *S*. Gallinarum contributes to the colonization of the gastrointestinal tract of chickens, as demonstrated with chickens orally infected by bacterial mutants [31], several genes encoding exported proteins are deleted in SPI-19 of the multi-hosts adapted *S*. Enteritidis, but were intact in the mammalian-adapted serovar *S*. Dublin (i.e. cattle and humans), as well as the avian-adapted serovars *S*. Gallinarum and *S*. Pullorum [22]. Among molecular mechanisms involved in fimbriae (i.e. *std.*∆, *stiC*, *stfF*, *safC*, *stbC*, *pegC*, *lpfC*, *sefD*, *sefC*, *sthB*, *sthA*, *sthE* in *S*. Gallinarum [25]), the partial losses of *ssf* and *saf* operons are highly specific to avian adaptation of *S*. Gallinarum and *S*. Pullorum [22].

Here, we propose a novel approach coupling identification of clade specific SNPs and InDels [1, 2] and associated gene-ontology enrichment analyses [14, 15] in order to link evolutionary history and biological function. By targeting the coregenome, we propose an additional and complementary method to analyses focusing on the accessory genome only. We tested its performance by studying the host-adaptation processes at the coregenome scale on a dataset constituted by Langridge et al. [22] in order to describe at the accessory genome scale the host adaptations of *Salmonella* serovars: Enteritidis (i.e. multi-hosts), Dublin (i.e. mammalian-hosts), Gallinarum (i.e. avian-hosts) and Pullorum (i.e. avian-hosts) [19, 22, 25].

## Results

### Robustness of the variant dataset

After trimming and mapping of paired-end reads, the previously published genome dataset (Additional file 1) displayed satisfactory depth (i.e. 175X) and breadth (i.e. 99 ± 1%) coverage (Additional file 2) against the reference genome *S.* Enteritidis (strain P125109) [32]. This depth coverage (i.e. >30X) is sufficient to accurately detect InDels with GATK [32], and this breadth coverage is quantitatively in accordance with results obtained by mapping of *S.* Typhimurium reads against the reference LT2 genome (i.e. 95% mean, 91% min., 98% max.) [24].

The set of sequencing data corresponding to 59 *Salmonella* genomes was analyzed through the variant calling workflow ‘VARCall’ where high-confidence variants were retained [33]. We identified 14,086 coregenome variants among the four serovars corresponding to 12,929 SNPs and 1157 InDels. Even if InDels are considered more challenging to be called accurately than SNPs [34], the ratio of 0.9 InDels/10 SNPs identified by the ‘VARCall’ workflow on the current *Salmonella* dataset falls inside the boundaries of 0.5–2 InDels / 10 SNPs which have been previously observed in other prokaryotic species (e.g. *Blochmannia vafer* [35], *Streptococcus* [36], *Salmonella* Typhimurium [24]) and eukaryotic studies (e.g. Rice [37], e.g. Human [38]).

We found that 12,031 variants (11,285 SNPs and 746 InDels) were intragenic, corresponding to 85% of the 14,086 coregenome variants (12,929 SNPs and 1157 InDels). As the coding regions represent about 88% of the *S.* Enteritidis chromosome (strain P125109, accession NC_011294.1) [25], intra and intergenic regions of the chromosome seem to be similarly impacted by variants. As observed by Zhou et al. [4] with 37% of intergenic InDels (i.e. 37/100) across 73 genomes of *S.* Agona serovar, we also detected 35% of intergenic InDels (i.e. 411/1157) across the 59 studied genomes.

**Fig. 1:**
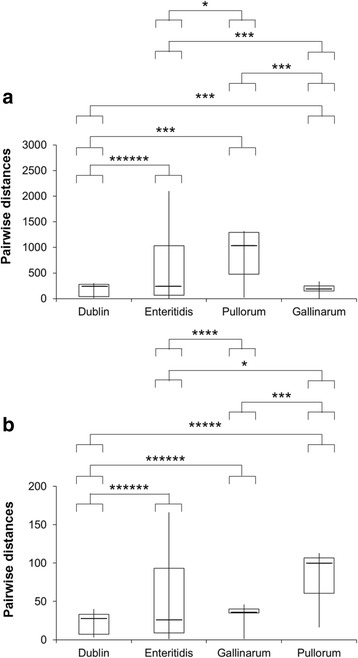
Boxplots (median, 25^th^ percentile, 75^th^ percentile, minimum and maximum) of pairwise distances expressed as single nucleotide polymorphisms (SNPs) (**a**) or small insertions/deletions (InDels) (**b**) into *Salmonella enterica* subsp. *enterica* serovars Dublin (*n* = 60), Enteritidis (*n* = 528), Pullorum (*n* = 10) and Gallinarum (*n* = 28). Normality of distribution and equality of variances were checked with Shapiro-Wilk and Fisher tests, respectively. Statistical differences (*: *p* < 5.0×10^−2^; **: *p* < 1.0×10^−2^; ***: *p* < 1.0×10^−3^; ****: *p* < 1.0×10^−4^; *****: *p* < 1.0×10^−5^; ******: *p* < 1.0×10^−6^) are calculated with Wilcoxon rank sum (i.e. non-normal distribution with equality of variances) or Kolmogorov-Smirnov (i.e. non-normal distribution without equality of variances) tests

Interestingly, the average pairwise distances of SNPs or InDels were significantly different (Fig. 1) between all the combinations of studied serovars (*p* < 5.0×10^−2^, Wilcoxon rank sum or Kolmogorov-Smirnov tests). For instance, the average pairwise distances of *S.* Enteritidis clade (531 ± 512 SNPs; 51 ± 48 InDels) were significantly different than those calculated for Pullorum (894 ± 501 SNPs; 84 ± 34 InDels), Gallinarum (195 ± 78 SNPs; 35 ± 9 InDels) and Dublin (163 ± 120 SNPs; 21 ± 13 InDels) serovars (*p* < 5.0×10^−2^, Wilcoxon rank sum or Kolmogorov-Smirnov tests).

### Robustness of the phylogenetic inference

**Fig. 2:**
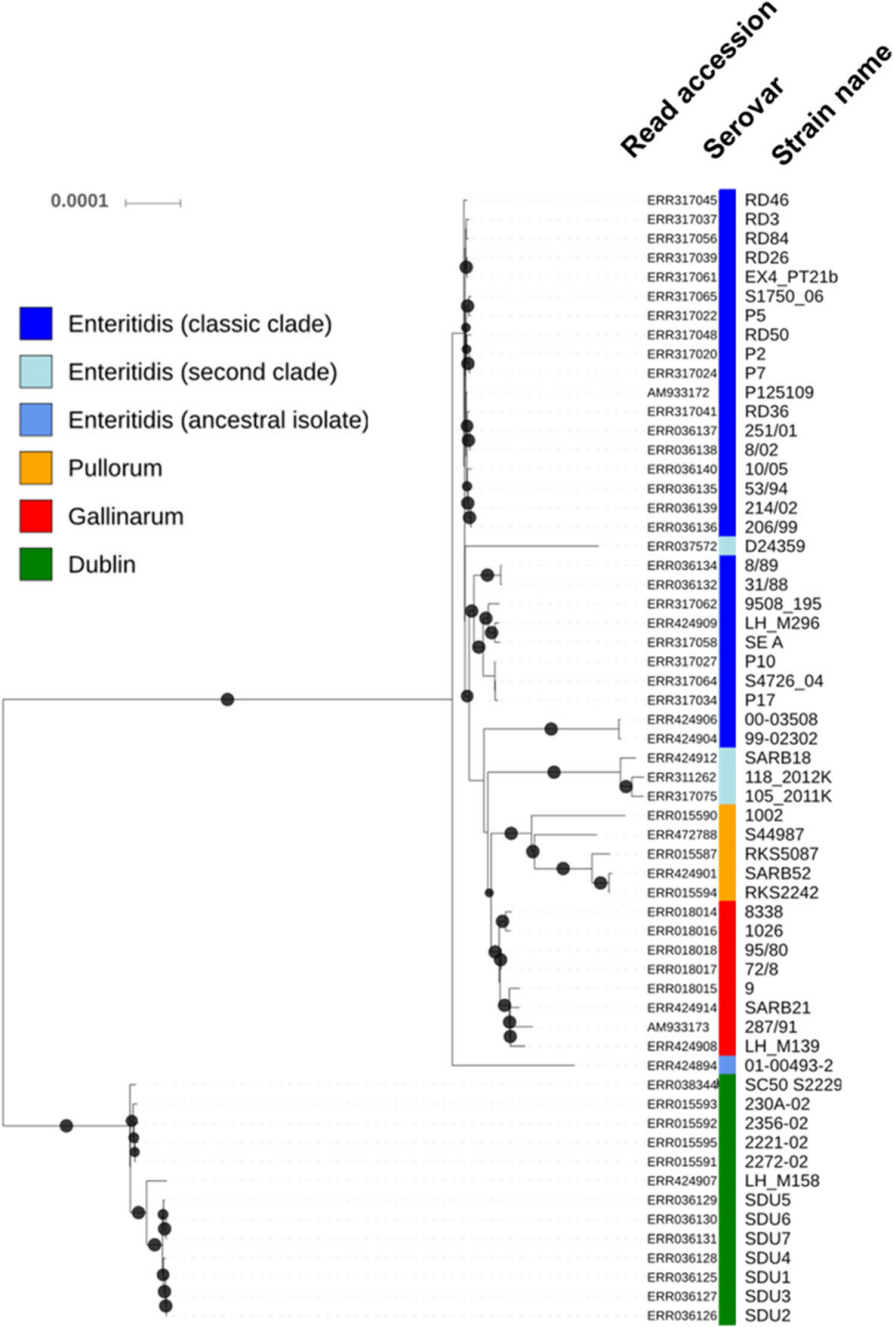
Phylogenetic inference based on coregenome single nucleotide polymorphisms (SNPs) identified in *Salmonella enterica* subsp. *enterica* serovars Dublin, Enteritidis, Pullorum, and Gallinarum. The color legend corresponds to serovars presented by Langridge et al. (Proc. Natl. Acad. Sci. 2015;112:863–8). The variants were identified by the ‘VARCall’ workflow against the reference genome *S.* Enteritidis (strain P125109, accession NC_011294.1). The produced pseudogenomes (4,685,848 bp) were inferred with RAxML based on a bootstrap analysis and search for best-scoring Maximum Likelihood tree with General Time-Reversible model of substitution and the secondary structure 16-state model. Bootstraps higher than 90% are represented by black circles. The phylogenetic inference converged after 200 bootstrap replicates with a log likelihood score of −8.10^6^ for 1000 computed trees. The tree is rooted on the branch of *S.* Dublin

The phylogenetic inference converged after 200 bootstrap replicates [39] with a log likelihood score of −8.10^6^ for 1000 computed trees [40], resulting in best-scoring Maximum Likelihood tree with most of branches presenting bootstraps higher than 90% (Fig. 2) and emphasizing the robustness of the tree reconstructed with SNPs detected by the ‘VARCall’ workflow.

The phylogenetic tree (Fig. 2) showed the mammalian-adapted serovar *S.* Dublin as a distinct clade with differences of 4945 ± 378 SNPs and 296 ± 17 InDels from the *S.* Enteritidis clade, as confirmed by our genetic structure analysis [41] and according to Langridge et al. [22]. Excepting few differences in terms of genome composition, our phylogenetic inferences excluding or including SNPs from recombination events (Additional file 3) showed two clades in *S.* Enteritidis described as classic and second clades by Langridge et al. [22] (Fig. 2). In addition to previous studies distinguishing these two clades of *S.* Enteritidis by microarray [19] and SNP-based approach [22], we supported also this dichotomy by a recombination event of 414 bp (node K in Additional files 4 and 5) harboring genes (POS 2018948–2,019,361) involved in DNA-binding (SEN_RS23380) and unknown function (SEN_RS09975).

### Host adaptation is not associated with a reduced coregenome diversity

*S.* Pullorum and *S.* Gallinarum are highly specialized toward avian-hosts; they are non-motile members of serogroup D, indistinguishable by serotyping and historically recognized as distinct biotypes considering several criteria: the distinct septocaemic diseases they cause in avian species, biochemical characteristics and multilocus enzyme electrophoresis [42]. As previously shown by Langridge et al. [22], our phylogenetic inference indicates that *S.* Pullorum and *S.* Gallinarum isolates arose from the second clade of *S.* Enteritidis (Fig. 2). Interestingly the pairwise distances of SNPs or InDels inside the two groups were significantly different (Fig. 1): the *S.* Gallinarum clade was homogeneous (195 ± 78 SNPs; 35 ± 9 InDels), while by contrast the *S.* Pullorum clade displayed a larger significant diversity with regards to SNPs (894 ± 501; *p* = 4.6×10^−13^; Fisher test) or InDels (84 ± 34; *p* = 1.4×10^−7^; Fisher test). These results confirm at the genetic level the biochemical classification performed by Crichton and Old [43].

All together, these observations are in accordance with Langridge et al. [22] who concluded that *S.* Dublin and *S.* Enteritidis have a most recent common ancestor, and that the clade *S.* Pullorum/*S.* Gallinarum diverged from *S.* Enteritidis. According to Langridge et al. [22], we also confirmed that the genome ERR424894 is an ancestral isolate of *S.* Enteritidis (Fig. 2 and Additional file 4). In opposite to studies theorizing that gains of large genetic elements by horizontal gene transfer would expand large host range of *S. enterica* subsp. *enterica* serovars, while the reduction of host range would mainly be explained by losses of genes due to large deletions and accumulation of pseudogenes [18], we observed that the coregenome diversities of the multi-hosts adapted serovar *S.* Enteritidis (Fig. 1), expressed in SNPs or InDels, were not significantly different than those of the avian-adapted serovar *S.* Pullorum (SNPs: *p* = 0.985; InDels: *p* = 0.262; Fisher test), as well as significantly higher than those of the mammalian-adapted serovar *S.* Dublin and avian-adapted serovar *S.* Gallinarum (SNPs: *p* < 4.6×10^−13^; InDels: *p* < 7.9×10^−15^; Fisher test).

### Accumulation of coregenome recombination events in the ancient branches

**Fig. 3:**
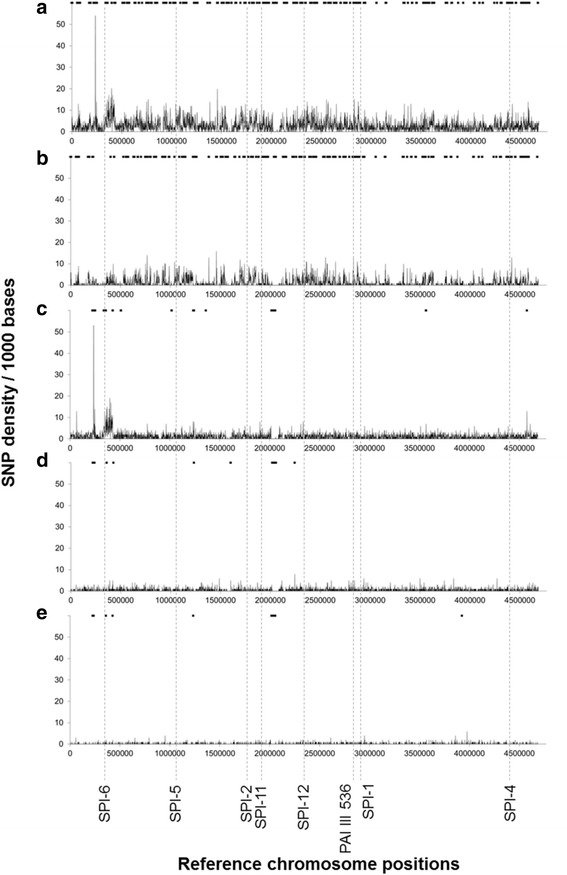
Densities of single nucleotide polymorphisms (SNPs) per 1000 bp (curves), *Salmonella* pathogenic islands (dotted lines), and recombination events (rectangles) across *Salmonella enterica* subsp. *enterica* serovars (**a**: 59 genomes, 12,929 SNPs), including Dublin (**b**: 13 genomes, 5084 SNPs), Enteritidis (**c**: 33 genomes, 5136 SNPs), Pullorum (**d**: 5 genomes, 2225 SNPs), and Gallinarum (**e**: 8 genomes, 671 SNPs). Pathogenicity island database from KonKuk University (Seoul, South Korea) were used to detect *Salmonella* Pathogenic Islands (SPIs) SPI-1 (2890501–2,934,879), SPI-2 (1727425–1,769,273), SPI-4 (4333507–4,361,514), SPI-5 (1053174–1,074,167), SPI-6 (299796–330,890), SPI-11 (1904313–1,912,607), SPI-12 (2328077–2,347,757) and PAI III 536 (2801306–2,810,695) of the reference genome *S.* Enteritidis (strain P125109, accession NC_011294.1)

Following lateral transfer of genetic material, recombination events are a common modality of evolution of bacterial genomes [44]. In *Salmonella*, the PAIs called SPIs have long been recognized to play a determinant role in the virulence properties of the pathogen [45]. These regions are likely acquired by lateral transfer and can be excised from the chromosome by site-specific recombination events [46]. We performed a specific recombination analysis on the whole dataset to identify branch specific recombination events that could explain some characteristics of the corresponding isolates. Detecting high densities of SNPs (Fig. 3), we identified 112 recombination events associated to 12 nodes of the phylogenetic inference (Additional files 4 and 5).

Surprisingly, most of these recombination events (*n* = 91) were detected in *S.* Dublin, the most distant clade of the collection (node A in Additional files 4 and 5). On the other hand, only 21 recombination signatures were detected in the 11 other nodes of the phylogenetic inference, indicating that recombination events are rare among the Enteritidis/Pullorum/Gallinarum serovars. A similar observation was made for the Agona serovar [4]. Certain recombination events were clade-specific, like those detected at nodes E and H, which are specific for *S.* Gallinarum or *S.* Enteritidis/*S.* Pullorum/*S.* Gallinarum, respectively (Additional files 4 and 5). For instance, the recombination event specific to *S.* Gallinarum impacted a segment of 418 bp harboring genes involved in the synthesis of multidrug resistance proteins (SEN_RS19005, SEN_RS19010).

We also detected two recombination events at node H where *S.* Enteritidis/*S.* Pullorum/*S.* Gallinarum split from *S.* Dublin (Additional files 4 and 5). This *S.* Enteritidis/*S.* Pullorum/*S.* Gallinarum (node H in Additional file 4) specific recombination events concerns a 4012 bp DNA sequence corresponding to genes encoding the FhuBCD ATP-dependent iron transport system and a 67,080 bp region carrying genes involved in the production of fimbrial proteins and transport of multidrug (Additional files 4 and 5).

### Phylogenetically relevant fixed variants

With the general aim to link functional information to the genetic variations identified by the ‘VARCall’ workflow, we retrieved the SNPs and InDels which are specific and sensitive (i.e. fixed variants) either of branches in the phylogenetic tree (i.e. ‘PhyloFixedVar’) or between groups defined by the user (i.e. ‘FixedVar’).

**Fig. 4:**
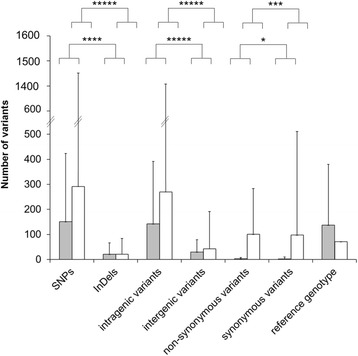
Homoplastic (grew bars) and non-homoplastic (white bars) variants (SNPs *versus* InDels, intragenic *versus* intergenic, non-synonymous *versus* synonymous) fixed across all branches of the phylogenetic inference including genomes of *Salmonella enterica* subsp. *enterica* serovars Enteritidis (*n* = 33), Pullorum (n = 5), Gallinarum (*n* = 8) and Dublin (*n* = 13). The variant annotation was performed with SnpEff against reference genome *S.* Enteritidis (strain P125109, accession NC_011294.1). The fixed non-homoplastic variants are defined by common genotypes across the considered group of genomes, as well as different genotypes in all the others compared genomes. The fixed homoplastic variants are defined by common genotypes across the considered group of genomes and genomes of independent phylogenetic clades, as well as different genotypes in genomes of the compared child-branches. The term ‘reference genotype’ refers to fixed variants presenting genotype of the reference genome. This analysis was performed with the script ‘phyloFixedVar’ (i.e. dependently of the phylogenetic inference). Statistical differences (*: *p* < 1.0×10^−6^; **: *p* < 1.0×10^−7^; ***: *p* < 1.0×10^−8^; ****: *p* < 1.0×10^−9^; *****: *p* < 1.0×10^−10^) are calculated with Wilcoxon signed rank tests. The vertical bars represent the standard deviation

The ‘phyloFixedVar’ and ‘FixedVar’ scripts retrieve SNPs and InDels and distinguish intragenic from intergenic variants, as well as homoplastic and non-homoplastic fixed variants at each branch of the phylogenetic tree. Additional information related to impact on protein translation was associated to each fixed variant (e.g. synonymous, non-synonymous, missense, frameshift). According to a study on *S.* Typhimurium estimating non-synonymous SNPs at 44% [24], most of the branches presented significantly more synonymous fixed variants that non-synonymous fixed variants (Fig. 4) with regard to non-homoplastic (*p* < 1.0×10^−8^; Wilcoxon signed rank test) variants (Fig. 4).

Focusing on branches having accumulated coregenome variants during evolution of *S.* Dublin, *S.* Enteritidis, *S.* Gallinarum and *S.* Pullorum (Fig. 2 and Additional file 4), most of fixed variants were identified at the node where *S.* Dublin and *S.* Enteritidis diverged (Table 1), as expected and whatever the considered type of variant (SNPs or InDels, intragenic or intragenic, homoplastic or non-homoplastic). For instance, fixed non-homoplastic InDels appeared in *S.* Enteritidis during the divergence with *S.* Dublin in two small intergenic regions between genes encoding a 4-hydroxy-2-oxo-heptane-1,7-dioate aldolase and a 4-hydroxyphenylacetate permease, or genes encoding a tripeptide permease A and a glutathione S-transferase, as well as in a gene encoding the fimbrial outer membrane usher protein SthC (Additional file 6).

**Table 1:** Single nucleotide polymorphisms (SNPs) and small insertions/deletions (InDels) fixed at phylogenetic branches where genomes of *Salmonella enterica* subsp. *enterica* serovars Enteritidis (*n* = 33), Pullorum (*n* = 5), Gallinarum (*n* = 8) and Dublin (*n* = 13) diverged

	Serovars	Variants							Total
		Intragenic					Intergenic		
		sSNP	nsSNP	nsInDel	rSNP	rInDel	SNP	InDel	
Homoplastic	Dublin *versus* all ^a^	0	0	0	0	0	0	0	0
	Enteritidis *versus* Dublin ^b^	0	0	0	3948	93	439	117	4597
	Pullorum + Gallinarum *versus* Enteritidis ^b^	0	0	0	0	0	0	2	2
	Pullorum *versus* Gallinarum ^a^	1	1	5	6	113	4	47	117
	Gallinarum *versus* Pullorum	0	0	8	236	84	16	38	382
Non-homoplastic	Dublin versus all ^a^	3129	819	87	0	0	438	115	4588
	Enteritidis *versus* Dublin ^b^	0	0	0	0	1	0	2	3
	Pullorum + Gallinarum *versus* Enteritidis ^b^	0	0	31	0	0	0	5	36
	Pullorum *versus* Gallinarum ^a^	95	139	81	0	0	15	27	357
	Gallinarum *versus* Pullorum ^a^	5	1	108	0	0	3	38	155

The variant calling analysis was performed with the ‘VARCall’ workflow (i.e. 12,929 SNPs and 1157 small InDels). The fixed non-homoplastic variants are defined by common genotypes across the considered group of genomes, as well as different genotypes in all the others compared genomes. The fixed homoplastic variants are defined by common genotypes across the considered group of genomes and genomes of independent phylogenetic clades, as well as different genotypes in genomes of the compared child-leaves. According to the variant annotation performed with SnpEff against reference genome *S.* Enteritidis (strain P125109, accession NC_011294.1), the fixed variants presenting reference (r) and alternative (synonymous: s; non-synonymous: ns) genotypes are presented

^a^Analysis performed with the script ‘phyloFixedVar’ (i.e. dependently of the phylogenetic inference)

^b^Analysis performed with the script ‘FixedVar’ (i.e. independently of the phylogenetic inference)

Considered in this case as homoplastic variants, the divergence of the clade Pullorum/Gallinarum with *S.* Enteritidis is also supported by these two InDels impacting these two small intergenic regions between pairs of genes encoding a 4-hydroxy-2-oxo-heptane-1,7-dioate aldolase and a 4-hydroxyphenylacetate permease, or a tripeptide permease A and a glutathione S-transferase (Table 1 and Additional file 6). Compared to *S.* Gallinarum, only one homoplastic and non-synonymous SNP has been fixed in *S.* Pullorum (Table 1) impacting a gene coding an hypothetical protein, as well as five InDels impacting genes encoding a type I secretion system permease/ATPase, an effector protein YopJ, a TonB-dependent receptor, a membrane protein and a 5-keto-4-deoxyuronate isomerase (Additional file 6). In comparison with *S.* Pullorum, only one non-homoplastic and non-synonymous SNP was fixed in *S.* Gallinarum impacting a gene coding a ribonuclease Z, as well as three non-homoplastic SNPs impacting two intergenic regions between pairs of genes coding a transcriptional regulator and a NAD^+^ synthetase, or an hypothetical protein and an α-glucosidase (Table 1 and Additional file 6).

Because several tens or hundreds of variants are fixed at these branches of interest (Table 1), we developed a tool in order to perform the first gene-ontology enrichment analyses based on sensitive and specific variants which are defined in the present study as intragenic and non-homoplastic fixed variants.

### Gene-ontology enrichment analyses

Gene-ontology enrichment analysis was developed to identify relevant biological processes associated to large number of biological objects [10–12]; our aim in the present work was to link fixed variants to GO-terms.

In order to retrieve biological functions impacted during evolution of the studied serovars, we performed four gene-ontology enrichment analyses: *S.* Dublin compared to all the 3 other serovars (Ontology 1 called ‘Dub_All’), divergence of *S.* Pullorum/Gallinarum from *S.* Enteritidis (Ontology 2 called ‘Ent_Pull/Gall’), divergences of *S.* Pullorum (Ontology 3 called ‘Pull_Gall’) and *S.* Gallinarum (Ontology 4 called ‘Gall_Pull’) with each other (Table 1). Our analysis focused on 4035, 31, 315 and 114 intragenic and non-homoplastic fixed variants specific of the ‘Dub_All’, ‘Ent_Pull/Gall’, ‘Pull_Gall’ and ‘Gall_Pull’ divergences, respectively; leading to the retrieval of 1841, 195, 1034, 1034 GO-terms that were significantly enriched (Additional file 7). The enriched GO-terms of the ‘Dub_All’ divergence represented biological process (54%) and cellular component (40%), while those of the other analyses corresponded to biological process (61 ± 3%) and molecular function (32 ± 3%) (Additional file 7). For further analysis we selected the most relevant GO-terms based on accuracy (i.e. GO-level ≥ 5), number of hits (i.e. ≥ 4) and *p*-value (i.e. < 5.0×10^-2^) (Table 2). Through this selection process we reduced the number of highly relevant GO-terms to 39, 2, 8, and 8 for ‘Dub_All’, ‘Ent_Pull/Gall’, ‘Pull_Gall’ and ‘Gall_Pull’ ontologies, respectively.

**Table 2:** Gene-ontology (GO) terms of intragenic and non-homoplastic variants (SNPs and InDels) fixed in *Salmonella enterica* subsp. *enterica* serovars Dublin *versus* all the others genomes (Ontology 1 called ‘Dub_All’), Pullorum/Gallinarum *versus* Enteritidis (Ontology 2 called ‘Ent_Pull/Gall’), Pullorum *versus* Gallinarum (Ontology 3 called ‘Pull_Gall’), and Gallinarum *versus* Pullorum (Ontology 4 called ‘Gall_Pull’)

Gene-ontology enrichment analysis	GO ID	GO-term	Number of hits	Expected number of hits	GO-level	*p*-value	Corrected *p*-value	Ontology
1	GO:0006105	succinate metabolic process	36	14.692	8	5.8×10^−13^	8.1×10^−10^	BP
	GO:0006307	DNA dealkylation involved in DNA repair	4	1.433	9	1.0×10^−42^	1.0×10^−39^	BP
	GO:0006468	protein phosphorylation	61	40.850	8	3.9×10^−05^	5.5×10^−02^	BP
	GO:0006520	cellular amino acid metabolic process	533	445.766	7	6.4×10^−08^	9.0×10^−05^	BP
	GO:0006525	arginine metabolic process	104	53.392	10	8.4×10^−18^	1.1×10^−14^	BP
	GO:0006527	arginine catabolic process	62	25.800	11	1.3×10^−19^	1.9×10^−16^	BP
	GO:0006545	glycine biosynthetic process	5	1.792	11	1.0×10^−42^	1.0×10^−39^	BP
	GO:0006560	proline metabolic process	81	46.583	10	2.4×10^−10^	3.4×10^−07^	BP
	GO:0006562	proline catabolic process	27	12.183	11	3.4×10^−08^	4.9×10^−05^	BP
	GO:0009064	glutamine family amino acid metabolic process	190	116.817	9	4.6×10^−17^	6.5×10^−14^	BP
	GO:0009065	glutamine family amino acid catabolic process	89	37.983	10	2.4×10^−25^	3.4×10^−22^	BP
	GO:0009233	menaquinone metabolic process	27	13.258	6	9.0×10^−07^	1.2×10^−03^	BP
	GO:0009234	menaquinone biosynthetic process	27	13.258	7	9.0×10^−07^	1.2×10^−03^	BP
	GO:0010133	proline catabolic process to glutamate	27	12.183	11	3.4×10^−08^	4.9×10^−05^	BP
	GO:0019544	arginine catabolic process to glutamate	10	3.942	12	1.2×10^−05^	1.7×10^−02^	BP
	GO:0019545	arginine catabolic process to succinate	36	14.692	9	5.8×10^−13^	8.1×10^−10^	BP
	GO:0035510	DNA dealkylation	4	1.433	8	1.0×10^−42^	1.0×10^−39^	BP
	GO:1,901,565	organonitrogen compound catabolic process	162	112.517	5	3.8×10^−09^	5.3×10^−06^	BP
	GO:1,901,605	alpha-amino acid metabolic process	320	240.441	8	8.0×10^−11^	1.1×10^−07^	BP
	GO:1,901,606	alpha-amino acid catabolic process	100	62.350	9	1.9×10^−09^	2.7×10^−06^	BP
	GO:0009379	Holliday junction helicase complex	4	1.408	5	1.0×10^−42^	1.0×10^−39^	CC
	GO:0003842	1-pyrroline-5-carboxylate dehydrogenase activity	27	12.957	6	1.5×10^−07^	1.8×10^−04^	MF
	GO:0003908	methylated-DNA-[protein]-cysteine S-methyltransferase activity	4	1.524	7	1.0×10^−42^	1.0×10^−39^	MF
	GO:0004020	adenylylsulfate kinase activity	4	1.524	6	1.0×10^−42^	1.0×10^−39^	MF
	GO:0004072	aspartate kinase activity	6	2.286	6	1.0×10^−42^	1.0×10^−39^	MF
	GO:0004372	glycine hydroxymethyltransferase activity	5	1.905	6	1.0×10^−42^	1.0×10^−39^	MF
	GO:0004412	homoserine dehydrogenase activity	6	2.286	6	1.0×10^−42^	1.0×10^−39^	MF
	GO:0004657	proline dehydrogenase activity	27	12.957	5	1.5×10^−07^	1.8×10^−04^	MF
	GO:0004743	pyruvate kinase activity	10	3.811	6	1.0×10^−42^	1.0×10^−39^	MF
	GO:0004815	aspartate-tRNA ligase activity	5	1.905	7	1.0×10^−42^	1.0×10^−39^	MF
	GO:0008770	[acyl-carrier-protein] phosphodiesterase activity	4	1.524	7	1.0×10^−42^	1.0×10^−39^	MF
	GO:0009015	N-succinylarginine dihydrolase activity	12	4.954	6	3.5×10^−06^	4.1×10^−03^	MF
	GO:0009017	succinylglutamate desuccinylase activity	10	4.192	6	2.4×10^−05^	2.8×10^−02^	MF
	GO:0015166	polyol transmembrane transporter activity	12	4.573	6	1.0×10^−42^	1.0×10^−39^	MF
	GO:0015169	glycerol-3-phosphate transmembrane transporter activity	12	4.573	8	1.0×10^−42^	1.0×10^−39^	MF
	GO:0015430	glycerol-3-phosphate-transporting ATPase activity	6	2.286	9	1.0×10^−42^	1.0×10^−39^	MF
	GO:0015605	organophosphate ester transmembrane transporter activity	12	4.573	5	1.0×10^−42^	1.0×10^−39^	MF
	GO:0016749	N-succinyltransferase activity	5	1.905	7	1.0×10^−42^	1.0×10^−39^	MF
	GO:0018480	5-carboxymethyl-2-hydroxymuconic-semialdehyde dehydrogenase activity	8	3.049	6	1.0×10^−42^	1.0×10^−39^	MF
2	GO:0003973	(S)-2-hydroxy-acid oxidase activity	4	0.013	6	1.1×10^−12^	1.3×10^−09^	MF
	GO:0016899	oxidoreductase activity, acting on the CH-OH group of donors, oxygen as acceptor	4	0.013	5	1.1×10^−12^	1.3×10^−09^	MF
3	GO:0043603	cellular amide metabolic process	36	17.451	5	1.9×10^−05^	2.7×10^−02^	BP
	GO:0006428	isoleucyl-tRNA aminoacylation	4	0.445	11	4.7×10^−05^	6.7×10^−02^	BP
	GO:0006522	alanine metabolic process	4	0.376	10	1.8×10^−05^	2.5×10^−02^	BP
	GO:0009078	pyruvate family amino acid metabolic process	4	0.376	9	1.8×10^−05^	2.5×10^−02^	BP
	GO:0004822	isoleucine-tRNA ligase activity	4	0.475	7	6.5×10^−05^	7.5×10^−02^	MF
	GO:0015079	potassium ion transmembrane transporter activity	8	1.680	9	3.7×10^−05^	4.2×10^−02^	MF
	GO:0008079	translation termination factor activity	5	0.657	7	3.0×10^−05^	3.4×10^−02^	MF
	GO:0003747	translation release factor activity	5	0.657	8	3.0×10^−05^	3.4×10^−02^	MF
4	GO:0043603	cellular amide metabolic process	36	17.473	5	2.0×10^−05^	2.8×10^−02^	BP
	GO:0006428	isoleucyl-tRNA aminoacylation	4	0.445	11	4.8×10^−05^	6.7×10^−02^	BP
	GO:0006522	alanine metabolic process	4	0.377	10	1.8×10^−05^	2.5×10^−02^	BP
	GO:0009078	pyruvate family amino acid metabolic process	4	0.377	9	1.8×10^−05^	2.5×10^−02^	BP
	GO:0004822	isoleucine-tRNA ligase activity	4	0.475	7	6.5×10^−05^	7.5×10^−02^	MF
	GO:0015079	potassium ion transmembrane transporter activity	8	1.681	9	3.7×10^−05^	4.2×10^−02^	MF
	GO:0008079	translation termination factor activity	5	0.658	7	3.0×10^−05^	3.4×10^−02^	MF
	GO:0003747	translation release factor activity	5	0.658	8	3.0×10^−05^	3.4×10^−02^	MF

The identification of variants, detection of fixed variants, assignment of GO-terms to variants, and gene-ontology enrichment analysis were performed with the scripts ‘VARCall’, ‘phyloFixedVar’, ‘GetGOxML’, and ‘EveryGO’, respectively. The level, biological process (BP), molecular function (MF), and cellular component (CC) of GO-terms are represented. The p-values of hypergeometric tests were adjusted by Bonferroni correction. The lowest corrected p-values representing GO-terms highly impacted by fixed variants (i.e. < 5.0×10^-2^), the highest GO-levels presenting the most accurate GO-terms (i.e. ≥ 5), and the highest number of hits representing relevant GO-terms quantitatively (i.e. ≥ 4) are presented

With only 3 non-homoplastic InDels fixed in two intergenic regions and in a gene coding the fimbrial outer membrane usher protein SthC (Table 1 and Additional file 6), the multi-hosts adapted serovar *S.* Enteritidis can be considered as a polyphyletic clade including the avian-adapted serovars *S.* Pullorum and *S.* Gallinarum (Fig. 2 and Additional file 4).

The *S.* Dublin monophyletic clade diverged from the *S.* Enteritidis polyphyletic clade (Fig. 2 and Additional file 4) by accumulating 4035 specific fixed variants (Table 1). These variants were found to be associated with 39 GO-terms mainly involved in central metabolism pathways and especially those of amino acids metabolism (Table 2). Hence, among these 39 GO-terms, 23 were directly related to amino acid metabolism and more specifically the catabolic processes of proline and arginine to glutamate (Table 2).

A similar analysis performed on the avian adapted serovars *S.* Pullorum and *S.* Gallinarum showed that the 31 fixed intragenic and non-homoplastic fixed variants (Table 1) impacted preferentially genes involved in oxidoreductase activity (Table 2). Interestingly, the unique non-homoplastic InDel fixed in all the genomes of *S.* Pullorum and *S.* Gallinarum (Table 1 and Additional file 6) disrupts the gene encoding FAD dependent oxidoreductase (POS 3067326, WP_000271927). This FAD dependent oxidoreductase also called glycolate oxidase (GOX) is an oxidoreductase that oxidizes α-hydroxy acids to α-keto acids with reduction of oxygen to H_2_O_2_. Its role remains elusive in eubacteria but its inactivation in the avian-restricted strains suggests that its activity is not compatible with an efficient colonization of the avian gut.

Emphasizing a biphyletic divergence, the 315 and 114 intragenic and non-homoplastic fixed variants differentiating *S.* Pullorum from *S.* Gallinarum (Table 1) impacted GO-terms related to various metabolic processes. However, the results indicate clear trends to accumulate modification in regions related to translation (i.e. 8/16 GO-terms), alanine and pyruvate metabolism, as well as potassium transport (Tables 2 and 3).

**Table 3:** Impacts of translation and function of proteins encoded by genes presenting GO-terms highly impacted by intragenic and non-homoplastic fixed variants in *Salmonella enterica* subsp. *enterica* serovars Pullorum and Gallinarum

GO	Position	Genes	Reference genotype	Genotype in Gallinarum	Genotype in Pullorum	Protein translation impact in Gallinarum	Protein translation impact in Pullorum	Impact on protein function
GO:0006428	54,044	SEN_RS00235	G	G	T	Null	Missense variant	Modification in Pullorum
GO:0006428	54,289	SEN_RS00235	T	T	C	Null	Synonymous variant	Potential modification
GO:0006428	54,658	SEN_RS00235	C	C	T	Null	Synonymous variant	Potential modification
GO:0006428	55,063	SEN_RS00235	G	G	A	Null	Synonymous variant	Potential modification
GO:0006522	1,313,705	SEN_RS06395	C	C	T	Null	Stop gained	Partial lost in Pullorum
GO:0006522	1,313,706	SEN_RS06395	A	A	G	Null	Missense variant	Modification in Pullorum
GO:0004822	54,044	SEN_RS00235	G	G	T	Null	Missense variant	Modification in Pullorum
GO:0004822	54,289	SEN_RS00235	T	T	C	Null	Synonymous variant	Potential modification
GO:0004822	54,658	SEN_RS00235	C	C	T	Null	Synonymous variant	Potential modification
GO:0004822	55,063	SEN_RS00235	G	G	A	Null	Synonymous variant	Potential modification
GO:0015079	99,941	SEN_RS00445	C	CGCTGGG	C	Disruptive inframe insertion	NULL	Partial lost in Gallinarum
GO:0015079	3,489,575	SEN_RS17095	C	CG	C	Frameshift variant	NULL	Modification in Gallinarum
GO:0003747	1,343,057	SEN_RS06530	C	C	A	Null	Synonymous variant	Potential modification
GO:0003747	1,343,237	SEN_RS06530	A	A	G	Null	Synonymous1 variant	Potential modification
GO:0003747	339,779	SEN_RS01530	G	G	T	Null	Missense variant	Modification in Pullorum

The identification of variants, detection of fixed variants, assignment of GO-terms to variants, and gene-ontology enrichment analysis were performed with the scripts ‘VARCall’, ‘phyloFixedVar’, ‘GetGOxML’, and ‘EveryGO’, respectively. The variant annotation was performed with SnpEff against reference genome *S.* Enteritidis (strain P125109, accession NC_011294.1). The p-values of hypergeometric tests were adjusted by Bonferroni correction. The lowest corrected p-values representing GO-terms highly impacted by fixed variants (i.e. < 5.0×10^-2^), the highest GO-levels presenting the most accurate GO-terms (i.e. ≥ 5), and the highest number of hits representing relevant GO-terms quantitatively (i.e. ≥ 4) are presented

### *Amino acid pathways related to divergence between S.* Enteritidis *and S.* Dublin

**Fig. 5:**
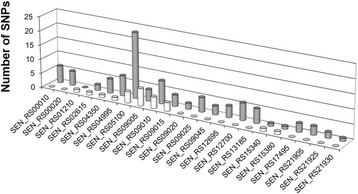
Genes impacted by single nucleotide polymorphisms (SNPs), involved in the amino acid catabolism, and fixed at the branch representing divergence between *Salmonella* serovars Dublin and Enteritidis/Pullorum/Gallinarum. Round bars represent missense (white) and synonymous SNPs (grew)

**Fig. 6:**
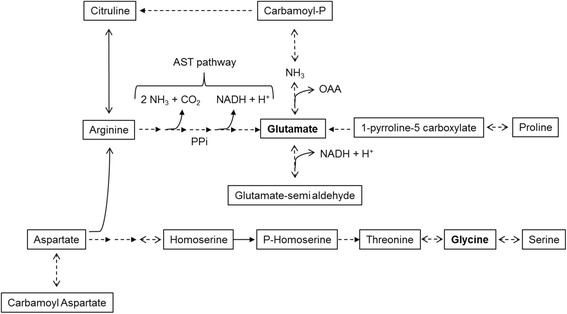
Amino acid pathways in which intragenic and non-homoplastic fixed single nucleotide polymorphisms (SNPs) differentiating *Salmonella* serovars Dublin *versus* Enteritidis have been detected. The dotted lines represent enzymatic steps for which the corresponding genes encoding enzymes have been specifically mutated. AST, NADH, OAA and PPi stand for ammonia-producing arginine succinyltransferase, nicotinamide adenine dinucleotide, oxaloacetic acid and pyrophosphate, respectively. KEGG database were used as a database for reference pathway (Nucl. Acids Res. 2016;44:D457–62)

The present analysis revealed that an important number of fixed variants differentiating *S.* Dublin from *S.* Enteritidis *sensu lato* have been accumulated into genes related to amino acid metabolism (Table 2). A more detailed analysis was then undertaken with a list of 22 genes directly involved in amino acid metabolism and in which 123 SNPs specific to *S.* Dublin have been fixed during the evolution process (Fig. 5). These 123 SNPs fall into the synonymous (81%) or missense (19%) categories. It is significant that none of these 123 mutations led to frameshift or interruption of reading frame suggesting that the corresponding gene functions were conserved. However, the biological consequences of these fixed SNPs are unclear at this stage. All the concerned genes encode enzymes which are involved in a series of reactions that we extracted using metabolic databases [47]. The Fig. 6 reports one of the most significant reconstructed metabolic network encompassing 12 genes among the 22 and shows that glutamate was a highly connected node.

### *Salmonella* pathogenic islands

Contrary to Langridge et al. who emphasized that evolution of *Salmonella* pathogenicity is strongly associated with the acquisition SPI-6 and SPI-19 [22], the present study identified intragenic and non-homoplastic fixed variants retained for the four presented gene-ontology enrichment analyses in SPI-1, SPI-2, SPI-4, SPI-5, SPI-6 and PAI III 536 (Additional file 8), as well as recombination evens impacting SPI-1, SPI-2, SPI-5 and SPI-12 (Additional file 9). While intragenic and non-homoplastic fixed variants impacting SPIs define common evolutionary traits of *S.* Dublin, *S.* Pullorum/Gallinarum, *S.* Pullorum and *S.* Gallinarum, we did not observed that SPIs impacted by recombination events are associated with these serovars (Fig. 3).

## Discussion

### Implementation of a workflow for gene-ontology enrichment analysis based on bacterial coregenome variants

Our objective to retrieve functional information from evolutionary relationships between genomes required first to build a tool to reconstruct robust phylogenetic inferences. The ‘VARCall’ workflow allows accurate qualitative and quantitative detection of coregenome SNPs and InDels to compute robust downstream phylogenetic inference. Because it is not possible to discriminate between absences of sequencing data and absences of sequences in samples, we did not take into account the variants when reads of at least one genome were missing in the alignments [6–9].

It must be emphasized that the number of coregenome variants is in accordance with the genetic distances between the genomes included in the variant calling analysis. Consequently, we recommend estimating genome pairwise distances before to run the ‘VARCall’ workflow in order to detect and exclude the potential divergent genomes which may cause a drastic fall in the coregenome variants during variant calling analysis. With this objective and independently of genome sizes, a pangenomic approach, combining *de novo* assemblies (SPAdes [48]) and estimations of Jaccard indexes with a form of locality-sensitive hashing of kmers (MinHash [49]), is currently under development in our team.

Although the removal of variants from recombination events must be theoretically performed when the phylogenetic inference assumes (i.e. Least squares, Minimum Evolution, Neighbor-Joining, UPGMA) or requires (i.e. Maximum Likelihood) a Markov chain model of nucleotide substitution [3], we observed according to Hedge and Wilson [50] that this removal induced a loss of information (Additional file 3), especially in depth branches where fixed variants from recombination events are a majority (Additional file 4).

Because of our objective to detect clade specific traits related to host adaptation, we decided to exclude the homoplastic variants in the analyses of the *S.* Dublin, *S.* Enteritidis, *S.* Gallinarum and *S.* Pullorum genomes. Considering all leaves of the phylogenetic inference, the non-homoplastic fixed variants represented 65% of all fixed variants. It should however be mentioned that it is fully possible to use the scripts ‘phyloFixedVar’, ‘FixedVar’, ‘GetGOxML’ and ‘EveryGO’ to select homoplastic variants for studies that would focus on coevolution [51].

The gene-ontology enrichment analysis was applied to synonymous and non-synonymous fixed variants because synonymous variants may be involved in regulation of gene expression or level of protein synthesis even if they do not impact the protein sequence [52].

In order to browse easily between tree branches, genes and their annotations, the xml file produced by the scripts ‘phyloFixedVar’ or ‘FixedVar’ centralizes the annotations of variants including the homoplastic variants for all the combinations of genomes present in the phylogenetic inference. The polyvalent scripts ‘phyloFixedVar’, ‘FixedVar’, ‘GetGOxML’ and ‘EveryGO’ were developed to perform the first gene-ontology enrichment analysis based on bacterial coregenome variants and can be applied to all kind of bacterial genome collection.

### Coregenome diversity is independent of host specialization in *Salmonella*

Contrary to studies showing that gene losses and accumulation of pseudogenes accompanied host specialization in bacteria [18], we did not observed that mammalian- and avian-adapted *Salmonella* displayed a decreasing in coregenome diversity (Fig. 1). However, and considering the high level of diversity of the *S.* Enteritidis coregenome, we may hypothesize that the MRCA of *S.* Enteritidis and *S.* Dublin was a multi-hosts adapted serovar because several studies concluded that the high genetic diversity of a multi-hosts adapted bacterial lineage represents a source for potential host specializations of less genetically diversified sub-lineages [53]. Because the reduction of the genome size mainly concerns endosymbiotic bacteria [54], we may also hypothesis that the divergence of the *Salmonella* sub-lineages is too recent to make any drastic decreasing of genome diversity related to host specializations observable.

### Transition from a free-living state to mammalian intestinal environment

With regards to fixed variants (Table 1), we focused on phylogenetic relevant variants defining the clades *S.* Dublin, *S.* Enteritidis, *S.* Pullorum and *S.* Gallinarum (Additional file 6). The fixed non-homoplastic InDel observed in the gene coding the fimbrial outer membrane usher protein SthC of the multi-hosts adapted serovar *S.* Enteritidis (i.e. fixed genotype ATT) may correspond to frameshift insertions of T or TT in *S.* Enteritidis, or frameshift deletions of T or TT in *S.* Dublin (Additional file 6). Both possibilities may be linked to adaptation to environment and gastrointestinal tracts of mammalian, respectively. The adaptation to free-living state is indeed known to be driven by adhesion on plants or biofilm formation which can be mediated by fimbrial proteins [55]. On the other hand the adaptation to the intestinal tracts of mammalian is known to be mediated by fimbriae operon during the transition from a free-living state to the intestinal environment, especially the *sth*ABCDE fimbrial operon which is frequent in non-typhoidal *Salmonella* serovars [56, 57] and required by *S.* Typhimurium for intestinal persistence in mice [58].

### The divergence between Dublin and Enteritidis targets regions involved in metabolic pathways of amino acids

The results of the present study highlighted the accumulation of fixed variants in intragenic regions connected to metabolic pathways of amino acids (Table 2). The most impacted pathway is the arginyl succinyl transferase pathway (AST pathway) involved in arginine catabolism. All 5 genes encoding the enzymes catalyzing the degradation of arginine to glutamate with the concomitant release of NH_3_, CO_2_ and regeneration of NADH + H^+^ accumulated mutations specific to each of the two serovars (Fig. 6). Concerning the GO-terms of interest related to metabolic and catabolism processes of amino acids in *S.* Dublin (Table 2 and Fig. 6), the glycine (GO:0006545, GO:0004372) may form serine or threonine but is not present in sufficient amounts to support growth of *S.* Typhimurium during intra host survival, and the glycine production or conversion of glycine to tetrahydrofuran (oxolane) are essential reactions during mice infection by *S.* Typhimurim [59]. Aerobic replication of *S.* Typhimurium in mice requires also the twin-arginine translocation system (Tat) which exports across the cytoplasmic membrane numerous cofactors containing virulence factors and anaerobic respiratory chain proteins [45] (GO:0006525, GO:0006527, GO:0019545, GO:0016749, GO:0009015). More precisely, with an interconnection between the biosynthesis of arginine and polyamines (i.e. putrescine and spermidine), the carbamoylphosphate is a precursor of arginine and is produced by the carbamoylphosphate synthetase (CPSase) with glutamine as the physiological amino group donor [60] (GO:0009064, GO:0009065, GO:0010133, GO:0019544, GO:0009017). The N-succinylarginine dihydrolase (gene *astB*) (GO:0009015) is indeed the second enzyme of the arginine succinyltransferase pathway involved in arginine catabolism as a sole nitrogen source [61].

Among its role as a protein component and as source of CO_2_ and NH_3_ through the GS/GOGA cycle, glutamate plays a major role in adaptation of bacteria to hyper osmotic conditions which are notably faced in the lumen of the digestive tract [62]. Our observations suggest a possible impact of the set of fixed SNPs on a differential glutamate accumulation capacity between *S.* Enteritidis and *S.* Dublin cells (Fig. 6). This hypothesis could be tested either by measuring cytoplasmic glutamate accumulation or growth under osmotic stress.

With regard to the GO-terms of interest for *S.* Dublin (Table 2 and Fig. 6), the accumulation of glutamate is correlated with *Salmonella* growth at high external osmolality [63] and aspartate is known to induce significant dysbacteriosis in gut microbiota of animals and humans [64]. Also included in GO-terms of interest for *S.* Dublin, the homoserine dehydrogenase (GO:0004412) is in fact a key enzyme in the biosynthetic pathway from aspartate to homoserine, which is a common precursor for the synthesis of amino acids methionine, threonine and isoleucine.

Regarding the proline metabolic and catabolic processes impacted by non-homoplastic fixed variants in *S.* Dublin (GO:0006560, GO:0006562, GO:0010133, GO:0004657, GO:0003842), hyperosmotic stress and proline limitation in host compartment is indeed known to lead *S.* Typhimurium responds to a decrease in the levels of proline-charged tRNA^Pro^ by promoting expression of the mgtCBR virulence operon [65]. Related to this observation, the 1-pyrroline-5-carboxylate dehydrogenase identified as GO-terms of interest for *S.* Dublin (GO:0003842) is encoded in the *putA* gene of *S.* Typhimurim by a bifunctional membrane-associated dehydrogenase which oxidizes proline to glutamate for use as the sole carbon, nitrogen or energy source [66].

The genes *alr* of *S.* Typhimurim, also called *dal* genes, encode alanine racemases which are biosynthesis sources of D-alanine for cell wall formation and also necessary to the catabolism of L-alanine as a source of carbon, energy and nitrogen [67]. L-arginine being used for growth of laying hens [68], the stop gained and missense variants in alanine racemase leading modifications of function in *S.* Pullorum (SEN_RS06395, POS 1313705 and POS 1313706, GO:0006522) may consequently be due to his adaptation to this avian alimentation.

Compared to similar sized nonflying mammalian, the avian gastrointestinal tracks present a typical shorter retention time and quantitatively much more important passive adsorption of L-glucose. During ontogeny, the architecture and functioning of mammalian and avian gastrointestinal tracts are closely related with their food diet and intake rate. The gestational phase of mammalian is dominated by production of gastrointestinal enzymes required for digestion and absorption of milk (e.g. amino acid transporters and the Na^+^-dependent D-glucose transporter SLGT1), whereas enzymes required for solid food and pinocytotic uptake capacity are produced during weaning (e.g. fructose and starch), and activities of sucrase-isomaltase, maltase-glucoamylase, trehalase and fructose transport (i.e. GLUT-5) increase when adult diet switches from lactose to sucrose and starch. In contrast, the pre- and post-natal periods of birds and chicks are associated with a switch of gastrointestinal functions driven by the transition from a lipid-rich yolk diet inside the egg (i.e. sucrase-isomaltase and the D-glucose transporter SLGT1) to a carbohydrate- and protein-based diet after hatch in young chickens and house sparrows (i.e. sucrose, maltase, maltase-glucoamylase and pancreatic amylase activities) [69]. Several specific amino acid transporters and a single proton-oligopeptide transporter (PEPT1) are responsible of the assimilation of protein components by the enterocytes of mammalian small intestines, but the specific amino acid transporters and role in protein nutrition of the ancient PEPT1 are still poorly described in avian [70].

### Acid survival in mammalian implies modifications of amino acids pathways

Further with respect to the GO-terms of interest related to metabolic and catabolism processes of amino acids in *S.* Dublin (Table 2 and Fig. 6), *S.* Typhimurium has also an active arginine-dependent arginine mechanism permitting survival at pH 2.5 [71]. *Salmonella* uses the tolerance response of low gastric pH and the arginine decarboxylase (gene *adiA*) acid resistance system to prepare for the stresses of host-cell interactions [72].

### Antibiotic resistances in avian implies fitness restorations

Mutations related to resistance acquisition and fitness restoration in the gene *ileS* encoding the essential enzyme isoleucine-tRNA ligase (IleRS) were observed in *S.* Enteritidis under selection pressure induced by the mupirocin, an inhibitor of this enzymatic activity [73]. Even if the mupirocin is not a common antibiotic for poultry farming, the missense variant in isoleucine-tRNA ligase leading modifications of function in *S.* Pullorum (SEN_RS00235, POS 54044, GO:0006428 + GO:0004822) may consequently also be linked to other antibiotics (Table 3).

### Limited ion supply in avian tract implies modifications of ion transport

Concerning the loss or modification of potassium ion transmembrane transporter activity in the avian-adapted serovar *S.* Gallinarum (GO:0015079) (Table 3), the modifications of ion biosynthesis is indeed also known to contribute to avian adaptation of *S.* Kentucky (e.g. turkey farms, Hatchery chicks, commercial broiler, layer flocks, commercial broiler environments, broiler processing plants, retail poultry products). For instance, plasmids encoding aerobactin (i.e. *iucABCD* and *iutA*) and Sit iron transport operons (i.e. *sitABCD*), as well as other iron acquisition genes (e.g. *iss*) play indeed a major role in survival abilities of *S*. Kentucky (e.g. IncFIB plasmid) and some *S*. Heidelberg (e.g. pSH163_120 and pSH696_117 plasmids) in the extraintestinal environments of poultry where iron is in limited supply [74].

## Conclusions

In conclusion, we proposed the first validated procedure to identify fixed SNPs and InDels according to inferred phylogenetic clades and performed the associated gene-ontology enrichment analysis in order to describe the adaptation of *Salmonella* serovars Dublin (i.e. mammalian-hosts), Enteritidis (i.e. multi-hosts), Pullorum (i.e. avian-hosts) and Gallinarum (i.e. avian-hosts) at the coregenome scale. Among the multiple metabolic pathways impacted by fixed variants during host adaptation of *Salmonella* serovars, our main observation emphasized that glutamate metabolism could play a major role in adaptation of *S.* Dublin to mammalian-hosts.

## Methods

### Read dataset

With the objective to validate the method of phylogenetic inference described in detail below, and to perform the first gene-ontology enrichment analysis based on bacterial coregenome variants, a previously published read dataset was used [22]. This collection of reads is made of 59 genomes sequences of *Salmonella* strains and was constituted by Langridge et al. [22] in order to describe host adaptation at the accessory gene scale of *Salmonella* serovars *S.* Enteritidis (i.e. multi-hosts), *S.* Dublin (i.e. mammalian-hosts), *S.* Gallinarum (i.e. avian hosts) and *S.* Pullorum (i.e. avian-hosts) (Additional file 1). While Langridge et al. proposed manual workflows mainly focusing on the accessory genome, we provide in the present manuscript automated workflows aiming to perform the first gene-ontology enrichment analysis based on bacterial coregenome variants.

### Variant calling analysis (i.e. the ‘VARCall’ workflow)

**Fig. 7:**
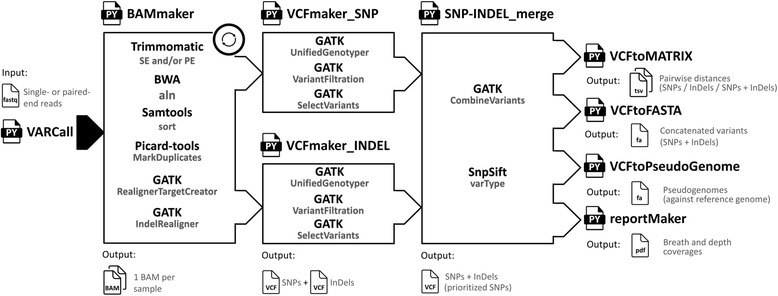
Programs (i.e. black letters) and commands (i.e. grew letters) implemented in the ‘VARCall’ workflow aiming to call single nucleotide polymorphisms (SNPs) and small insertions/deletions (InDels). The scripts referring to alignment against reference genome (i.e. ‘BAMmaker’), variant calling (i.e. ‘VCFmaker_SNP’ and ‘VCFmaker_INDEL’), variant combination (i.e. ‘SNP-INDEL_merge’), pairwise distances (i.e. ‘VCFtoMATRIX’), variant concatenation (i.e. ‘VCFtoFASTA’), pseudogenome assemblies (i.e. ‘VCFtoPseudoGenome’), and report about breadth and depth coverages (i.e. ‘reportMaker’) were written with Python 2.7 and are invoked by the driven script ‘VARCall’ (i.e. black arrow). The script ‘BAMmaker’ is performed for each genome (i.e. circular arrow)

A driver script called ‘VARCall’ invokes ‘BAMmaker’, ‘VCFmaker_SNP’, ‘VCFmaker_INDEL’, and ‘SNP-INDEL_merge’, successively (Fig. 7). The script ‘BAMmaker’ allows trimming of single- and paired-end reads with Trimmomatic (i.e. quality score of 25 and minimal length of 20 bp) [75], read alignment against a reference genome with BWA [76], read sorting with Samtools [77], as well as duplication removal and realignment around InDels with the Genome Analysis Toolkit (GATK) [2], successively. Following an approved framework for variant discovery [78], the scripts ‘VCFmaker_SNP’ and ‘VCFmaker_INDEL’ call and filter variants (i.e. SNPs and InDels) according to GATK best practices [2] in order to retain high-confidence variants [33]. After variant combination prioritizing SNPs with GATK [2] and SNPs/InDels flagging with SnpSift [79], the variants presenting allele frequencies equal to one across the dataset of genomes (i.e. variants specific to the reference genome), as well as the variants presenting missing genotypes in at least one genome, are removed from the dataset of variants (i.e. vcf file). The ‘VARCall’ workflow produces four output files: matrices of pairwise distances (‘VCFtoMATRIX), a report about breadth and depth coverages (i.e. script ‘reportMaker’), and files of concatenated variants (i.e. script ‘VCFtofasta’) and pseudogenomes (i.e. ‘VCFtoseudogenomes’) to build fast or slow phylogenetic inferences, respectively (Fig. 7). The pseudogenomes correspond to the reference genome where the genotypes of detected variants are replaced in each genome of the dataset. Finally, the variants are annotated with SnpEff (version 4.1 g without variants from intron, UTR-5’, UTR-3′, upstream regions, and downstream regions) [80] based on the reference genome annotation from NCBI (i.e. gbk file). Independently of the ‘VARCall’ workflow, the density of SNPs were computed with the ‘vcf-subset’ module and the ‘SNPdensity’ argument of VCFtools [81].

We develop currently a new version of the ‘VARCall’ workflow (i.e. iVARCall2) based on the HaplotypeCaller algorism (GATK) in order to call SNPs and InDels at the same time by local *de novo* assembly for each genome which would give us the opportunity to compute a faster phylogenetic inference based on variants independently called for each genome (HaplotypeCaller) rather than to perform time consuming variant calling across reference genome alignments (UnifiedGenotyper) [82].

### Phylogenetic inference

Based on the pseudogenomes, the phylogenetic inference was performed with the multi-core architecture of RAxML [40]. Following computation of a parsimony starting tree, a rapid bootstrap analysis and search was computed for best-scoring Maximum Likelihood (ML) tree with General Time-Reversible (GTR) model of substitution [83] and the secondary structure 16-state model (i.e. nwk file). A posterior bootstrap convergence test was performed in order to determine if sufficient bootstrap replicates were computed [39], and the log likelihood score of all trees was also computed with RAxML [40]. Based on the pseudogenomes and the RAxML phylogenetic inference, the recombination events were detected using ClonalFrameML locating regions with high densities of SNPs on each branch [5]. The positions of recombination events detected by ClonalFrameML were also removed from the vcf file with a script ‘Clonal_VCFilter’ in order to compute phylogenetic inference based on pseudogenomes excluding variants linked to recombination events. Finally, the best-scoring ML trees were graphically represented with iTOL viewer [84]. The comparison of the tree topologies was performed using the cophylo function of ‘phytools’ R package [85]. In order to support results of phylogenetic inference, genetic structure analyses were also performed with BAPS based on SNPs [41].

### Fixed and homoplastic variants

A script called ‘phyloFixedVar’ uses the annotated vcf file from the variant calling analysis and the nwk file from a binary tree. This script was developed in order to detect sensitive and specific variants at each branch of the phylogenetic inference. The variants are defined with a biological point of view either as fixed (i.e. variants with sensitive and specific genotypes at intermediate branches) or transient (i.e. variants with sensitive and specific genotypes at final leaves). The script ‘phyloFixedVar’ (a) identifies all comparisons of genome groups referring to all nodes, (b) selects sensitive and specific variants, and (c) defines if these variants are homoplastic (i.e. convergence of genotypes between independent phylogenetic clades), successively. More precisely, right child leaves are listed with corresponding leaves of left child at each node and reversely, then both comparisons (i.e. right versus left children and reversely) are connected with comparison numbers which are associated to identifiers of single nodes, all together grouped into node labels in a new nwk file (a). On the one hand the sensitive variants are detected as presenting common genotypes into leaves of right or left child of previously listed comparisons, and on the other hand the specific variants are identified as presenting different genotypes in corresponding right or left children (b). With regard to these sensitive and specific variants, the genotypes of all the other genomes are screened in order to tag homoplastic variants (i.e. common genotypes between variants of independent phylogenetic clades) (c). The selected variants are finally written in the xml file with their related annotations (i.e. genotype, effect of homoplasy, NCBI identifiers, gene IDs, gene names, type, position, phenotypical impact) for each node and all comparisons of lists of leaves. Similarly to ‘phyloFixedVar’, another script called ‘FixedVar’ requires a vcf file and lists of genomes IDs that have to be compared. This script was developed in order to detect sensitive and specific variants independently of the phylogenetic inference.

### Gene-ontology enrichment analysis

**Fig. 8:**
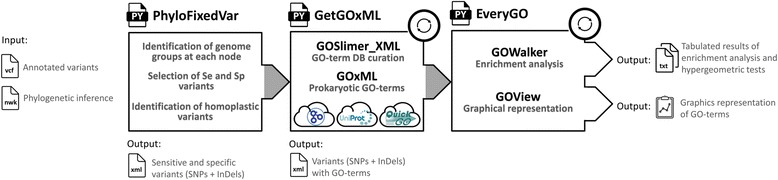
Programs (i.e. black letters) and corresponding effects (i.e. grew letters) implemented in the scripts ‘phyloFixedVar’, ‘GetGOxML’ and ‘EveryGO’ aiming to identify sensitive (Se) and specific (Sp) variants (SNPs and InDels) at each branches of corresponding phylogenetic inference, associate prokaryotic gene ontology (GO) terms with these homoplastic and/or non-homoplastic variants, and perform gene-ontology enrichment analysis based on the parent-child approach integrating hypergeometric tests and Bonferroni corrections, respectively. Online databases are queried by the scripts ‘GOSlimer’ and ‘GOxML’ (i.e. clouds). The GO database of the Gene Ontology Consortium is used by the script ‘GOSlimer’ to identify prokaryotic GO-terms. The QuickGO browser of the UniProt GO annotation program is queried by the script ‘GOxML’ to associate the variants with the corresponding GO-terms. These scripts were written with Python 2.7 and implement R libraries ‘p.ajust’ and ‘phyper’. The whole workflow is semi-automated (i.e. grew arrows) and the scripts ‘GetGOxML’ and ‘EveryGO’ can be performed for each variant and each branch, respectively (i.e. circular arrow)

With the objective to associate the selected variants to corresponding prokaryotic GO-terms, a driver script called ‘GetGOxML’ invokes the scripts ‘GOSlimer_XML’ and ‘GOxML’, successively (Fig. 8). More precisely, the script ‘GOSlimer_XML’ aims to generate lists of prokaryote GO-terms based on the GO database of the Gene Ontology Consortium [14] (i.e. go-basic.obo file: http://geneontology.org/page/download-ontology), and the script ‘GOxML’ associates gene identifiers (i.e. NP or WP) from the xml file with GO-terms available in the QuickGO browser (http://www.ebi.ac.uk/GOA) of the UniProt GO annotation program (https://www.ebi.ac.uk/QuickGO/). In addition, the script ‘GOxML’ allows comparison of these GO-terms in order to exclude potential eukaryote GO-terms from the dataset and retains prokaryote GO-terms. Finally, the script ‘GOxML’ integrates the curated GO-terms (i.e. prokaryote GO-terms) and related biological processes to the common xml file in order to centralized the GO-terms and functional annotations of variants (i.e. genotype, effect of homoplasy, NCBI identifiers, gene IDs, gene names, type, position, phenotypical impact). With a view to select intragenic variants (SNPs and InDels), and distinguish between GO-terms from the variants of interest (i.e. tested sample) and all variants (i.e. universe) which are used for the hypergeometric test of the gene-ontology enrichment analysis based on the parent-child approach [15], the driver script ‘EveryGO’ invokes ‘GOWalker.R’ and ‘GOView.R’, successively (Fig. 8). More precisely, the script ‘GOWalker.R’ counts the GO-terms from the sample (i.e. variants from compared leaves) and universe (i.e. all variants) for each GO-term, as well as the sizes of the sample (i.e. total GO-terms in the sample) and universe (i.e. total GO-terms in the universe). Then, the script ‘GOWalker.R” performs the hypergeometric test and Bonferroni correction [86] implemented in the ‘stats’ and ‘phyper’ R libraries, respectively [87]. Finally, the script ‘GOView.R’ aims to compute a graphical representation of the gene-ontology enrichment analysis with the plotting system ggplot2 (i.e. *p*-values of the hypergeometric tests *versus* the branch levels from the GO-terms of the DAG).

### *Salmonella* pathogenic islands

Candidates of PAI-like region overlapping genomic islands (cPAIs) of the Pathogenicity Island Database (http://www.paidb.re.kr) from KonKuk University (Seoul, South Korea) [88] were used to detect SPI-1 (2890501–2,934,879), SPI-2 (1727425–1,769,273), SPI-4 (4333507–4,361,514), SPI-5 (1053174–1,074,167), SPI-6 (299796–330,890), SPI-11 (1904313–1,912,607), SPI-12 (2328077–2,347,757) and PAI III 536 (2801306–2,810,695) according to the reference genome *S.* Enteritidis (strain P125109, accession NC_011294.1).

## Additional files

Additional file 1: Genome dataset used in the present study. The genomes of *Salmonella enterica* subsp. *enterica* serovars Enteritidis, Pullorum, Gallinarum and Dublin were described by Langridge et al. (Proc. Natl. Acad. Sci. 2015;112:863–8). (XLSX 15 kb)

Additional file 2: Statistical report of the ‘VARCall’ workflow. The serovars *Salmonella enterica* subsp. *enterica* serovars Dublin, Enteritidis, Pullorum and Gallinarum were described by Langridge et al. (Proc. Natl. Acad. Sci. 2015;112:863–8). (XLSX 16 kb)

Additional file 3: Phylogenetic inferences performed based on coregenome single nucleotide polymorphisms (SNPs) excluding (A) or including (B) variants from recombination events detected in *Salmonella enterica* subsp. *enterica* serovars Dublin, Enteritidis, Pullorum and Gallinarum. The variants were identified by the ‘VARCall’ workflow against the reference genome *S.* Enteritidis (strain P125109, accession NC_011294.1). The positions of recombination events detected by Maximum Likelihood and default gamma priors of ClonalFrameML are removed with a script ‘Clonal_VCFilter’ in order to compute phylogenetic inference based on pseudogenomes excluding variants linked to recombination events. The produced pseudogenomes (4,685,848 bp) were inferred with RAxML based on a bootstrap analysis and search for best-scoring Maximum Likelihood tree with General Time-Reversible model of substitution and the secondary structure 16-state model. The color legend corresponds to phylogenetic clustering performed by Langridge et al. (Proc. Natl. Acad. Sci. 2015;112:863–8). The trees are rooted on the branches of *S.* Dublin before comparison. The comparison of the tree topologies were performed using the cophylo function of ‘phytools’ R package. (PDF 1733 kb)

Additional file 4: Phylogenetic inference based on coregenome single nucleotide polymorphisms (SNPs) and recombination events identified in *Salmonella enterica* subsp. *enterica* serovars Dublin, Enteritidis, Pullorum and Gallinarum. The color legend corresponds to serovars presented by Langridge et al. (Proc. Natl. Acad. Sci. 2015;112:863–8). The variants were identified by the ‘VARCall’ workflow against the reference genome *S.* Enteritidis (strain P125109, accession NC_011294.1). The produced pseudogenomes (4,685,848 bp) were inferred with RAxML based on a bootstrap analysis and search for best-scoring Maximum Likelihood tree with General Time-Reversible model of substitution and the secondary structure 16-state model. The phylogenetic inference converged after 200 bootstrap replicates with a log likelihood score of −8.10^6^ for 1000 computed trees. The tree is rooted on the branch of *S.* Dublin. The pseudogenomes and the RAxML inference were used to perform detection of recombination events based on default gamma priors of ClonalFrameML. The number of recombination events is defined closed to white circles which represent recombination events occurred on a branch of the phylogenetic tree. The recombination events with sizes higher than 400 bp are presented. (PDF 752 kb)

Additional file 5: List of recombination events identified in *Salmonella enterica* subsp. *enterica* serovars Dublin, Enteritidis, Pullorum and Gallinarum. The variants were identified by the ‘VARCall’ workflow against the reference genome *S.* Enteritidis (strain P125109, accession NC_011294.1). The produced pseudogenomes (4,685,848 bp) were inferred with RAxML based on a bootstrap analysis and search for best-scoring Maximum Likelihood tree with General Time-Reversible model of substitution and the secondary structure 16-state model. The pseudogenomes and the RAxML inference were used to perform detection of recombination events based on default gamma priors of ClonalFrameML. The recombination events with sizes higher than 400 bp are presented. (XLSX 16 kb)

Additional file 6: Phylogenetically relevant single nucleotide polymorphisms (SNPs) and small insertions/deletions (InDels) fixed at phylogenetic branches where genomes of *Salmonella enterica* subsp. *enterica* serovars Enteritidis (*n* = 33), Pullorum (*n* = 5), Gallinarum (*n* = 8) and Dublin (*n* = 13) diverged. The variant calling analysis was performed with the ‘VARCall’ workflow (i.e. 12,929 SNPs and 1157 small InDels). The fixed non-homoplastic variants are defined by common genotypes across the considered group of genomes, as well as different genotypes in all the others compared genomes. The fixed homoplastic variants are defined by common genotypes across the considered group of genomes and genomes of independent phylogenetic clades, as well as different genotypes in genomes of the compared child-leaves. The variants were annotated with SnpEff against reference genome *S.* Enteritidis (strain P125109, accession NC_011294.1). (XLSX 13 kb)

Additional file 7: Gene-ontology (GO) terms of intragenic and non-homoplastic variants (SNPs and InDels) fixed in *Salmonella enterica* subsp. *enterica* serovars Dublin *versus* all the others genomes (Ontology 1 called ‘Dub_All’), Pullorum/Gallinarum *versus* Enteritidis (Ontology 2 called ‘Ent_Pull/Gall’), Pullorum *versus* Gallinarum (Ontology 3 called ‘Pull_Gall’), and Gallinarum *versus* Pullorum (Ontology 4 called ‘Gall_Pull’). The variant annotation was performed with SnpEff against reference genome *S.* Enteritidis (strain P125109, accession NC_011294.1). The identification of variants, detection of fixed variants, assignment of GO-terms to variants, and gene-ontology enrichment analysis were performed with the scripts ‘VARCall’, ‘phyloFixedVar’, ‘GetGOxML’, and ‘EveryGO’, respectively. The level, biological process (BP), molecular function (MF), and cellular component (CC) of GO-terms are represented. The *p*-values of hypergeometric tests were adjusted by Bonferroni correction. (XLSX 327 kb)

Additional file 8: Candidates of PAI-like region overlapping genomic islands (cPAIs) impacted by intragenic and non-homoplastic variants (SNPs and InDels) fixed in *Salmonella enterica* subsp. *enterica* serovars Dublin *versus* all the others genomes (Ontology 1 called ‘Dub_All’), Pullorum/Gallinarum *versus* Enteritidis (Ontology 2 called ‘Ent_Pull/Gall’), Pullorum *versus* Gallinarum (Ontology 3 called ‘Pull_Gall’), and Gallinarum *versus* Pullorum (Ontology 4 called ‘Gall_Pull’). Pathogenicity Island Database from KonKuk University (Seoul, South Korea) were used to detect *Salmonella* Pathogenic Islands (SPIs) SPI-1 (2890501–2,934,879), SPI-2 (1727425–1,769,273), SPI-4 (4333507–4,361,514), SPI-5 (1053174–1,074,167), SPI-6 (299796–330,890), SPI-11 (1904313–1,912,607), SPI-12 (2328077–2,347,757) and PAI III 536 (2801306–2,810,695) of the reference genome *S.* Enteritidis (strain P125109, accession NC_011294.1). (XLSX 251 kb)

Additional file 9: Candidates of PAI-like region overlapping genomic islands (cPAIs) impacted recombination events identified in *Salmonella enterica* subsp. *enterica* serovars Dublin, Enteritidis, Pullorum and Gallinarum. The variants were identified by the workflow ‘VARCall’ against the reference genome *S.* Enteritidis (strain P125109, accession NC_011294.1). The produced pseudogenomes (4,685,848 bp) were inferred with RAxML based on a bootstrap analysis and search for best-scoring Maximum Likelihood tree with General Time-Reversible model of substitution and the secondary structure 16-state model. The pseudogenomes and the RAxML inference were used to perform detection of recombination events based on default gamma priors of ClonalFrameML. The recombination events with sizes higher than 400 bp are presented. Pathogenicity Island Database from KonKuk University (Seoul, South Korea) were used to detect *Salmonella* Pathogenic Islands (SPIs) SPI-1 (2890501–2,934,879), SPI-2 (1727425–1,769,273), SPI-4 (4333507–4,361,514), SPI-5 (1053174–1,074,167), SPI-6 (299796–330,890), SPI-11 (1904313–1,912,607), SPI-12 (2328077–2,347,757) and PAI III 536 (2801306–2,810,695) of the reference genome *S.* Enteritidis (strain P125109, accession NC_011294.1). (XLSX 19 kb)

## Abbreviations

AST: Arginyl succinyl transferase
BP: Biological process
CC: Cellular component
cPAIs: Candidates of PAI-like region overlapping genomic islands
CPSase: Carbamoylphosphate synthetase
CRISPR: Clustered regularly interspaced short palindromic repeats
DAG: Directed acyclic graph
GLUT-5: fructose transport
GO: Gene ontology
GOX: Glycolate oxidase
IleRS: Isoleucine-tRNA ligase
InDels: small insertions/deletions
MF: Molecular function
MRCA: Most recent common ancestor
NADH: Nicotinamide adenine dinucleotide
OAA: Oxaloacetic acid
PAIs: Pathogenicity islands
PEPT1: Proton-oligopeptide transporter
PPi: Pyrophosphate
SLGT1: Na^+^-dependent D-glucose transporter
SNPs: Single nucleotide polymorphisms
SPIs: *Salmonella* pathogenicity islands
T6SSs: Type VI secretion systems
Tat: Twin-arginine translocation system
