# Supplementary material for: First gene-ontology enrichment analysis based on bacterial coregenome variants: insights into adaptations of Salmonella serovars to mammalian- and avian-hosts
Source: BMC Microbiol. 2017 Nov 28;17:222. doi: 10.1186/s12866-017-1132-1 (PMC5706153; doi:10.1186/s12866-017-1132-1)
Supplement: Supplementary file 3 — Phylogenetic inferences performed based on coregenome single nucleotide polymorphisms (SNPs) excluding (A) or including (B) variants from recombination events detected in Salmonella enterica subsp. enterica serovars Dublin, Enteritidis, Pullorum and Gallinarum. The variants were identified by the ‘VARCall’ workflow against the reference genome S. Enteritidis (strain P125109, accession NC_011294.1). The positions of recombination events detected by Maximum Likelihood and default gamma priors of ClonalFrameML are removed with a script ‘Clonal_VCFilter’ in order to compute phylogenetic inference based on pseudogenomes excluding variants linked to recombination events. The produced pseudogenomes (4,685,848 bp) were inferred with RAxML based on a bootstrap analysis and search for best-scoring Maximum Likelihood tree with General Time-Reversible model of substitution and the secondary structure 16-state model. The color legend corresponds to phylogenetic clustering performed by Langridge et al. (Proc. Natl. Acad. Sci. 2015;112:863–8). The trees are rooted on the branches of S. Dublin before comparison. The comparison of the tree topologies were performed using the cophylo function of ‘phytools’ R package. (PDF 1733 kb) [file 12866_2017_1132_MOESM3_ESM.pdf]

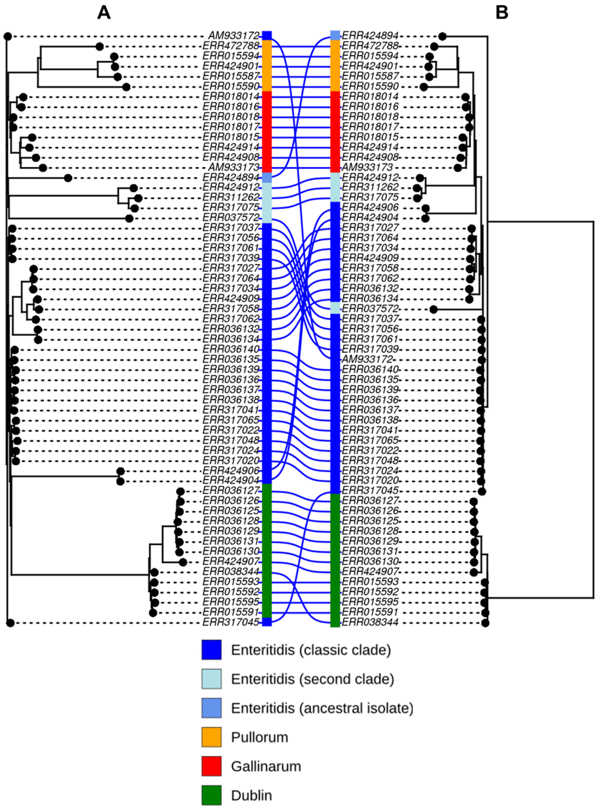

A
B
AM933172
ERR472788
ERR015594
ERR424901
ERR015587
ERR015590
ERR018014
ERR018016
ERR018018
ERR018017
ERR018015
ERR424914
ERR424908
AM933173
ERR424894
ERR424912
ERR311262
ERR317075
ERR037572
ERR317037
ERR317056
ERR317061
ERR317039
ERR317027
ERR317064
ERR317034
ERR424909
ERR317058
ERR317062
ERR036132
ERR036134
ERR036140
ERR036135
ERR036139
ERR036136
ERR036137
ERR036138
ERR317041
ERR317065
ERR317022
ERR317048
ERR317024
ERR317020
ERR424906
ERR424904
ERR036127
ERR036126
ERR036125
ERR036128
ERR036129
ERR036131
ERR036130
ERR424907
ERR038344
ERR015593
ERR015592
ERR015595
ERR015591
ERR317045
Enteritidis (classic clade)
Enteritidis (second clade)
Enteritidis (ancestral isolate)
Pullorum
Gallinarum
Dublin
