# Supplementary material for: First gene-ontology enrichment analysis based on bacterial coregenome variants: insights into adaptations of Salmonella serovars to mammalian- and avian-hosts
Source: BMC Microbiol. 2017 Nov 28;17:222. doi: 10.1186/s12866-017-1132-1 (PMC5706153; doi:10.1186/s12866-017-1132-1)
Supplement: Supplementary file 4 — Phylogenetic inference based on coregenome single nucleotide polymorphisms (SNPs) and recombination events identified in Salmonella enterica subsp. enterica serovars Dublin, Enteritidis, Pullorum and Gallinarum. The color legend corresponds to serovars presented by Langridge et al. (Proc. Natl. Acad. Sci. 2015;112:863–8). The variants were identified by the ‘VARCall’ workflow against the reference genome S. Enteritidis (strain P125109, accession NC_011294.1). The produced pseudogenomes (4,685,848 bp) were inferred with RAxML based on a bootstrap analysis and search for best-scoring Maximum Likelihood tree with General Time-Reversible model of substitution and the secondary structure 16-state model. The phylogenetic inference converged after 200 bootstrap replicates with a log likelihood score of −8.106 for 1000 computed trees. The tree is rooted on the branch of S. Dublin. The pseudogenomes and the RAxML inference were used to perform detection of recombination events based on default gamma priors of ClonalFrameML. The number of recombination events is defined closed to white circles which represent recombination events occurred on a branch of the phylogenetic tree. The recombination events with sizes higher than 400 bp are presented. (PDF 752 kb) [file 12866_2017_1132_MOESM4_ESM.pdf]

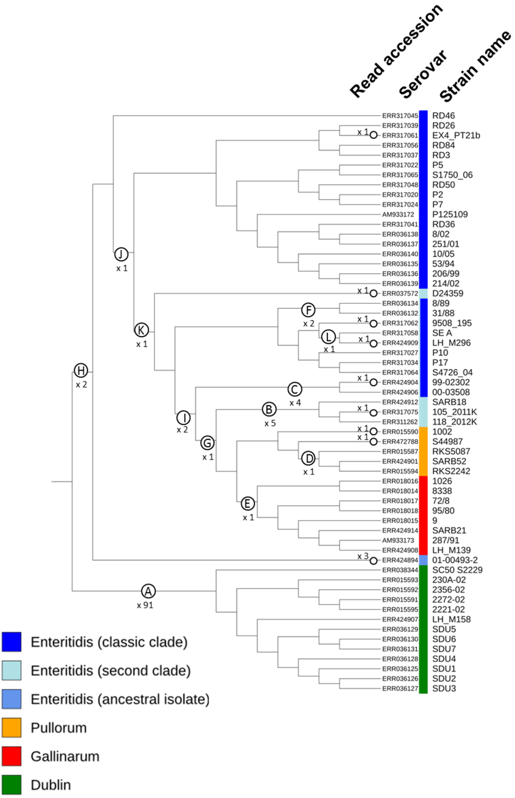
Read accession
Serovar
Strain name
ERR317045 RD46
ERR317039 RD26
ERR317061 EX4_PT21b
ERR317056 RD84
ERR317037 RD3
ERR317022 P5
ERR317065 S1750_06
ERR317048 RD50
ERR317020 P2
ERR317024 P7
AM933172 P125109
ERR317041 RD36
ERR036138 8/02
ERR036137 251/01
ERR036140 10/05
ERR036135 53/94
ERR036136 206/99
ERR036139 214/02
ERR037572 D24359
ERR036134 8/89
ERR036132 31/88
ERR317062 9508_195
ERR317058 SE A
ERR424909 LH_M296
ERR317027 P10
ERR317034 P17
ERR317064 S4726_04
ERR424904 99-02302
ERR424906 00-03508
ERR424912 SARB18
ERR317075 105_2011K
ERR311262 118_2012K
ERR015590 1002
ERR472788 S44987
ERR015587 RKS5087
ERR424901 SARB52
ERR015594 RKS2242
ERR018016 1026
ERR018014 8338
ERR018017 72/8
ERR018018 95/80
ERR018015 9
ERR424914 SARB21
AM933173 287/91
ERR424908 LH_M139
ERR424894 01-00493-2
ERR038344 SC50 S2229
ERR015593 230A-02
ERR015592 2356-02
ERR015591 2272-02
ERR015595 2221-02
ERR424907 LH_M158
ERR036129 SDU5
ERR036130 SDU6
ERR036131 SDU7
ERR036128 SDU4
ERR036125 SDU1
ERR036126 SDU2
ERR036127 SDU3
A x 91
B x 5
C x 4
D x 1
E x 1
F x 2
G x 1
H x 2
I x 2
J x 1
K x 1
L x 1
x 1
x 3
Enteritidis (classic clade)
Enteritidis (second clade)
Enteritidis (ancestral isolate)
Pullorum
Gallinarum
Dublin
